# Effect of Low-Temperature Pyrolysis on the Properties of Jute Fiber-Reinforced Acetylated Softwood Kraft Lignin-Based Thermoplastic Polyurethane

**DOI:** 10.3390/polym10121338

**Published:** 2018-12-03

**Authors:** Hyun-gyoo Roh, Sunghoon Kim, Jungmin Lee, Jongshin Park

**Affiliations:** Department of Biosystems and Biomaterials Science & Engineering, Seoul National University, 1 Gwanak-ro, Gwanak-gu, Seoul 08826, Korea; ssangno@snu.ac.kr (H.-g.R.); bv206driver@snu.ac.kr (S.K.); archon04@snu.ac.kr (J.L.)

**Keywords:** lignin, acetylated lignin, thermoplastic polyurethane, jute fiber, low-temperature pyrolysis, short-fiber-reinforced elastomers

## Abstract

Short jute fiber-reinforced acetylated lignin-based thermoplastic polyurethane (JF reinforced ASKLTPU) was prepared and characterized as a short-fiber-reinforced elastomer with carbon-neutrality and biodegradability. The acetylated softwood kraft lignin-based thermoplastic polyurethane (ASKLTPU) was prepared with polyethylene glycol (PEG) as a soft segment. Short jute fiber was modified using low-temperature pyrolysis up to the temperatures of 200, 250, and 300 °C in order to remove non-cellulosic compounds of jute fibers for enhancing interfacial bonding and reducing hydrophilicity with the ASKLTPU matrix. JF-reinforced ASKLTPUs with fiber content from 5 to 30 wt % were prepared using a melt mixing method followed by hot-press molding at 160 °C. The JF-reinforced ASKLTPUs were characterized for their mechanical properties, dynamic mechanical properties, thermal transition behavior, thermal stability, water absorption, and fungal degradability. The increased interfacial bonding between JF and ASKLTPU using low-temperature pyrolysis was observed using scanning electron microscopy (SEM) and also proved via interfacial shear strength measured using a single-fiber pull-out test. The mechanical properties, thermal properties, and water absorption aspects of JF-reinforced ASKLTPU were affected by increased interfacial bonding and reduced hydrophilicity from low-temperature pyrolysis. In the case of the degradation test, the PEG component of ASKLPTU matrix highly affects degradation and deterioration.

## 1. Introduction

Lignin is a kind of complex heterogeneous polymer with aromatic and aliphatic moieties and is the second most abundant natural biopolymers on earth [1,2]. Lignin is regarded as large volume renewable biomass or feedstock, which does not belong to the human chain, and has characteristics of carbon neutrality [3,4,5,6] and biodegradability by fungi [7,8]. Industrial lignin or technical lignin, such as lignosulfonate or kraft lignin, is separated as a by-product during the wood pulp and papermaking process. However, only 1–2% of lignin is used as specialty products, while most lignin is used as low-value fuel [2,9]. Therefore, many studies were conducted on lignin for high-value applications including fuels [10], carbonaceous source for carbon materials [5], component of copolymer such as polyurethane [11,12] and epoxies [13,14,15], adhesives [14,16,17], and polymer blends [14,18]. Especially, lignin-based copolymers, including polyurethane or epoxy resins, could be an environmentally-friendly alternative for commercial polymers due to its carbon neutrality and biodegradability [1,4,7,14].

Jute fiber is a kind of cellulosic fiber and often used for rope, yarn, cordage, textiles, paper products, and fiber reinforcement for composites [19]. As natural lignocellulosic fiber, jute has merits of relatively low density in comparison to glass fiber, high specific strength and modulus, biodegradability, and carbon-neutrality [20,21]. However, the relatively hydrophilic nature of natural fibers makes it undesirable to make natural fiber-reinforced polymer composites due to the hydrophobic polymer matrix [20,22]. It is known that containing more non-cellulosic compounds, such as hemicelluloses and waxes, in the biomass fibers increases the hydrophilicity and moisture absorptivity of the fiber [22,23,24]. Thus, the physical treatments, such as plasma treatment [25,26], and the chemical treatment, such as silane treatment [20] and alkaline treatment [27], are used for natural fiber modification.

Pyrolysis means the thermal decomposition or degradation of the biomass in the absence of oxygen and is used for fuels or other high-value products [28]. The dry torrefaction or low-temperature pyrolysis was used for the conversion from bulk biomass to an energy-dense solid as fuel [29]. In the previous work, low-temperature pyrolysis of jute fibers were studied for easier thermomechanical modification method [30]. Because the decomposition of three major compounds of biomass; cellulose, hemicellulose, and lignin were distinct, the low-temperature pyrolysis up to the temperature of 200, 250, and 300 °C could be able to remove non-cellulosic compounds, such as waxes and hemicelluloses, with the least damage to the cellulose component of jute fibers [30,31,32]. The reduction of non-cellulosic compounds decreases hydrophilicity of jute fibers and could increase interfacial bonding of jute fibers and the polymer matrix [30]. Other studies on natural fibers related to heat treatments had been limited to only relatively low temperatures (i.e., 150 °C) [33] or specific purposes (i.e., studying molding conditions) [34].

Thermoplastic polyurethane is a kind of thermoplastic elastomer with the structure of rubbery soft segments and separated hard domains [35]. Short fibers or nano-scale reinforcements were used for reinforcing thermoplastic polyurethane. Glass fibers [36,37], aramid fibers [38,39,40,41,42,43], carbon fibers [37,44], and nano-reinforcements [45,46] were used for reinforcing thermoplastic polyurethane. Natural fibers were also used to reinforce thermoplastic polyurethane in the view of the development of natural fiber-reinforced biocomposites [47,48,49,50,51,52,53,54]. However, the studies for bio-based thermoplastic polyurethane reinforced with natural fibers were limited, though it was expected to have characteristics of carbon-neutrality and biodegradability [55].

In the previous work, acetylated softwood kraft lignin-based thermoplastic polyurethane (ASKLTPU) were prepared and characterized [56]. ASKLTPU showed comparable mechanical properties to conventional thermoplastic polyurethane; however, the reinforcement was considered for further applications requiring elastomers with a relatively high modulus or strength. Meanwhile, it would be appropriate to use pyrolyzed jute fibers (PJF) in order to reinforce bio-based and/or biodegradable plastics or elastomers. The biocomposites reinforced using PJF would retain its carbon-neutrality and/or biodegradability in addition to improving the interfacial bonding by reducing inherent hydrophilicity of natural fibers using low-temperature pyrolysis. Thus, combining the aspects of ASKLTPU and PJF, the low-temperature pyrolyzed jute fiber-reinforced acetylated softwood kraft lignin-based thermoplastic polyurethane (PJF reinforced ASKLTPU) were prepared in order to study short-fiber-reinforced elastomer with carbon-neutrality and biodegradability. As mentioned above, the studies of natural fiber-reinforced bio-based TPU with both carbon-neutrality and biodegradability were rarely discussed [55]. The chemical nature, morphological aspects, and the various properties of PJF-reinforced ASKLTPU were characterized to investigate the short-fiber-reinforced elastomers from renewable resources. Furthermore, the properties of PJF reinforced ASKLTPU was also investigated in order to study the effect of low-temperature pyrolysis, which is rarely used for the modification of natural fiber reinforcement.

## 2. Materials and Methods 

### 2.1. Materials

Softwood kraft lignin (SKL) (Indulin AT^®^) was purchased from MeadWestvaco Co. (Richmond, VA, USA) and kept in the desiccator without moisture. Raw jute fibers (RJF; Bangladesh Jute Institute, Dhaka, Bangladesh), approximately 50–100 μm in diameter, were cut into 65 mm segments and kept at 15.8 ± 0.2 °C and 26 ± 2% RH (relative humidity). Acetic anhydride (99.0%), polyethylene glycol (PEG1000; *M*_n_ = 1000 g/mol; Samchun Chemical, Seoul, Korea), 4,4-methylene diphenyl diisocyanate (MDI, 97.0%; Kanto Chemical, Saitama, Japan), and tetrahydrofuran (THF, 99.5%; Junsei Chemical, Tokyo, Japan) were purchased and used. Sabouraud dextrose agar (model No: 210950) purchased from Becton, Dickinson and Company (BD, Franklin Lakes, NJ, USA) was used as a medium and *Aspergillus awamori* fungi (ATCC 6970) from Korean Collection for Type Cultures (KCTC, Jeongeup-si, Korea) were used for the degradation test.

### 2.2. Acetylation of Softwood Kraft Lignin (SKL)

SKL was acetylated according to the procedure described in the previous work [56,57]. A total of 100 g of SKL, dried under vacuum at 105 °C, was reacted with 300 mL of acetic anhydride at 80 °C for 40 min. Then, the reactants were quenched by ethanol/water mixture (1:1; *v*/*v*). The resultant solid, acetylated softwood kraft lignin (ASKL) was washed using deionized water, followed by vacuum drying at 105 °C for 24 h. The degree of acetylation of prepared ASKL was approximately 90% according to the previous work [56].

### 2.3. Preparation of ASKL-Based Thermoplastic Polyurethane (ASKLTPU30)

ASKL-based thermoplastic polyurethane (ASKLTPU30), whose ASKL content was 30 wt %, was prepared according to the previous works [56]. About 50 g of THF was added to the polyol mixture, which consisted of 10.5 g of vacuum dried ASKL and 18.81 g of vacuum dried PEG. The polyol mixture solution was stirred at 300 rpm for 20 min, followed by adding 5.68 g (–NCO/–OH ratio: 1.05) of MDI. The mixture was reacted for 5–20 min until the reactant became fairly thick. The reacting solution was cast onto a PTFE dish coated with a urethane mold release. The cast sheet was placed into a vacuum oven and degassed at 90 °C for 2 h, followed by curing at 90 °C for 48 h. Finally, the resultant ASKLTPU sheets were conditioned at room temperature. In addition, ASKLTPU10 and ASKLTPU20, whose ASKL content was 10 wt % and 20 wt %, respectively, were also prepared with the same procedure and a 1.05 –NCO/–OH ratio in order to use for further discussions.

### 2.4. Low-Temperature Pyrolysis of Jute Fiber

As a modification method, low-temperature pyrolysis of jute fiber was performed according to the previous work [30]. Jute fiber was put into the laboratory muffle furnace (LT3/11/P330, Nabertherm GmbH, Liliental, Germany). It was pyrolyzed with a heating rate of 5 °C/min from room temperature to up to 200 °C, 250 °C, and 300 °C, under nitrogen gas in the furnace. At the maximum pyrolysis temperature, the resultant low-temperature pyrolyzed jute fiber (PJF) was taken out without air-contact and quickly quenched. PJFs were labelled as PJF200, PJF250, and PJF300 after their maximum pyrolysis temperatures.

### 2.5. Preparation of JF Reinforced ASKLTPU

JF-reinforced ASKLTPU was prepared using a melt-mixing method followed by hot-press molding. RJF and PJFs (JF) as fiber components and ASKLTPU30 as a matrix component were vacuum-dried in 105 °C for 24 h before use. The fibers were chopped to approximately 10 mm lengths and ASKLTPU30 were mixed using a table-type kneader (PBV-0.3, Irie Shokai Co., Yokohama, Japan) at 160 °C, followed by a hot-press method using two post-manual hydraulic presses (#2699, Carver Inc., Wabash, IN, USA) at 160 °C. The fiber contents of the reinforced ASKLTPU were 5, 10, 20, and 30 wt %. JF reinforced ASKLTPU was labelled PJF200/ASKLTPU30 10/90, whose composition was 10 wt % of PJF200 and 90 wt % of ASKLTPU30. For comparison, ASKLTPU30P and ASKLTPU30 without fiber reinforcement were prepared using the same procedure.

### 2.6. Characterizations of JF-Reinforced ASKLTPU

#### 2.6.1. Fourier Transform Infrared (FTIR) Spectroscopy

Spectroscopic analysis of PJF-reinforced ASKLTPU was performed using attenuated total reflectance—Fourier transform infrared spectroscopy (ATR-FTIR) (Nicolet™ iS™ 5 FTIR Spectrometer with an iD5 ATR accessory, Thermo Scientific, Waltham, MA, USA) technique. The ATR-FTIR measurements were performed at room temperature in the range of 4000–650 cm^−1^ at a resolution of 0.5 cm^−1^ with 32 scans.

#### 2.6.2. Scanning Electron Microscopy (SEM) Analysis

A field emission scanning electron microscope (FE-SEM) (AURIGA, Carl Zeiss, Oberkochen, Germany) was used to observe the tensile fracture surfaces of PJF-reinforced ASKLTPU. The reinforced ASKLTPU were coated with a thin layer of platinum by sputter coater and visualized with a voltage of 2 kV.

#### 2.6.3. Single-Fiber Pull-Out Test

A single-fiber pull-out test was performed to measure interfacial shear strength (IFSS) between JF and ASKLTPU30 [58,59,60]. The samples for single-fiber pull-out test were prepared by embedding the single individual fiber of JF in ASKLTPU30 matrix (embedded length: about 0.8 mm) and hot-press molding (Figure S1). The prepared samples were subjected to a tensile force with speed of 1.0 mm/min using a universal testing machine (UTM) with a 10-N XLC series load cell (LRX-0500-A1, LLOYD Instruments Ltd., Bognor Regis, UK). The load required to pull the fiber out of the matrix was obtained from force-extension curve of the single-fiber pull-out test. The IFSS was calculated using the following formula:

IFSS = *F/πDL* (1)

where *F* is the load required to debond the fiber from the matrix, *D* is the diameter of the JF, and *L* is the embedded length of fiber into the matrix. At least five specimens were measured for each JF/ASKLTPU sample.

#### 2.6.4. Tensile Test

The mechanical properties of PJF-reinforced ASKLTPU was analyzed via a tensile test using a universal testing machine (UTM) with an XLC series load cell (LRX-0500-A1, LLOYD Instruments, Hampshire, UK). The tensile test was performed according to ASTM D 638-10 guidelines with ASTM D638 type V (dumbbell-shape) specimens, while some testing settings were modified due to characteristics of the samples [61]. The gage length, strain rate, and preload of tensile tests were set as 10.0 mm, 200 mm/min, and 0.1 N, respectively. All tests were performed at 23.0 °C and 45% RH.

#### 2.6.5. Frequency Distributions of Reinforcements

An optical microscope (Eclipse LV100, Nikon, Tokyo, Japan) was used to measure the length and the diameter of JFs in JF-reinforced ASKLTPU for a further modelling study of mechanical properties regarding the prediction of Young’s modulus. At least 120 specimens were observed to measure the frequency distributions.

#### 2.6.6. Dynamic Mechanical Thermal Analysis (DMA)

Dynamic mechanical thermal analysis (DMA) of PJF-reinforced ASKLTPU was performed using dynamic mechanical thermal analyzer (DMA/SDTA861e, Mettler Toledo, Greifensee, Switzerland). The samples were disk-shaped with a 6 mm diameter and about 2 mm in height. The storage and loss moduli were scanned using the compression mode in the temperature range from −50 to 200 °C at a heating rate of 3 °C/min with a purge of dry N_2_. The force amplitude was 3 N and the displacement amplitude was 20 μm, while the strain frequency was 1 Hz.

#### 2.6.7. Differential Scanning Calorimetry (DSC)

The thermal transition behaviors of JF-reinforced ASKLTPU were investigated using a differential scanning calorimeter (DSC 200 F3, Netzsch, Burlington, MA, USA). The analysis was performed in the range of −80–200 °C at heating and cooling rates of 10 °C/min with a purge of dry N_2_. The first cooling cycle and second heating cycle were investigated for thermal transition behavior. 

#### 2.6.8. X-ray Diffraction Analysis (XRD)

X-ray diffraction (XRD) analysis was performed in order to check the crystallinity of JF-reinforced ASKLTPU using X-ray Diffractometer (D8 ADVANCE with DAVINCI, BRUKER, Hamburg, Germany) with CuKα radiation (λ = 0.15406 nm) generated at 40 mA and 40 kV in the 2θ range 3–50° with a step size of 0.02.

#### 2.6.9. Thermogravimetric Analysis (TGA)

The thermogravimetric analyzer (TGA) (Q-5000 IR, TA Instruments Inc., New Castle, DE, USA) was used to investigate the thermal stability of JF-reinforced ASKLTPU30. Samples were analyzed in the range of 20–700 °C with filled nitrogen gas and the heating rate was 10 °C/min.

#### 2.6.10. Water Absorption

Water absorption tests of ASKLTPUs and JF-reinforced ASKLTPUs was conducted using a modified long-time immersion method based on the ASTM D570-98 standard [62]. The samples were dried in the heating oven at 60 °C for 24 h before immersion in deionized water. After drying, the samples were weighed and soaked in deionized water at room temperature. The samples were weighed to the nearest 0.001 g immediately after wiping off all surface water at specific time intervals after immersion. The average of three samples for each material type was used for analysis.

#### 2.6.11. Degradation Test

**Figure 1:**
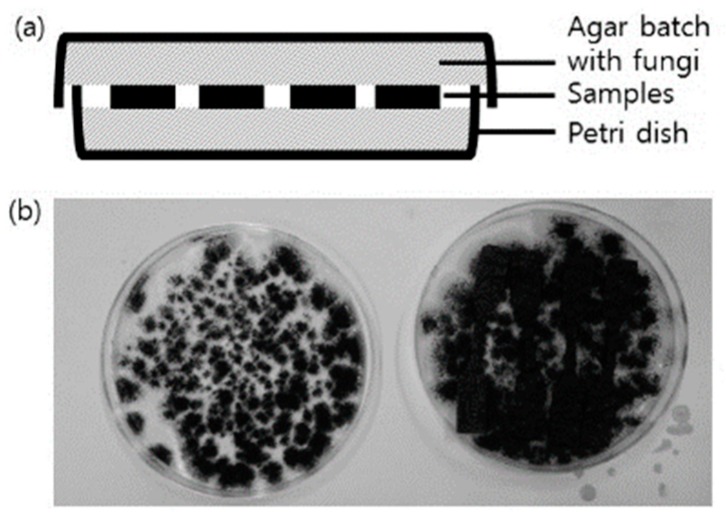
(**a**) The scheme of experimental settings for degradation test; (**b**) The picture of the fungi *Aspergillus awamori* grown as a batch and the placement of samples for the degradation test.

Degradation test of ASKLTPU10, ASKLTPU30, RJF/ASKLTPU30 10/90, and PJF250/ASKLTPU30 10/90 was performed to investigate the biodegradability of ASKLTPU and PJF-reinforced ASKLTPU according to the previous work [63]. The medium based on Sabouraud dextrose agar was prepared on the basis of the BD Difco’s manual [64] and subsequently sterilized in an autoclave. After coating 15 mL of the medium on both sides of a petri dish (90 mm diameter × 15 mm height), *Aspergillus awamori* fungi were seeded. The fungi in the medium were allowed to grow for 96 h at 25 °C and 80% RH. Afterwards, ASKLTPU and PJF-reinforced ASKLTPU samples in the form of the specimen for tensile test (ASTM D638 Type V) were placed on the middle of the medium coated surfaces in contact (Figure 1). The fungi were allowed to degrade the samples under the condition of 25 °C and 80% RH for 2, 4, 6, 8, and 16 weeks. After each selected period, the sample was taken out from the medium-coated surfaces and washed softly to remove attached fungi. The washed sample was stored in a desiccator for 24 h to remove the residual water. Finally, the change in mechanical properties were measured using the same procedure discussed above in a tensile test section and the weight loss of the sample was measured using following formula:

*Weight loss* (%) = ((*W*_0_ – *W*_1_)/*W*_0_) × 100% (2)

where *W*_0_ is the weight of the sample measured before the degradation and *W*_1_ is the weight measured after the degradation.

## 3. Results and Discussion

### 3.1. FTIR Spectroscopy Analysis

**Figure 2:**
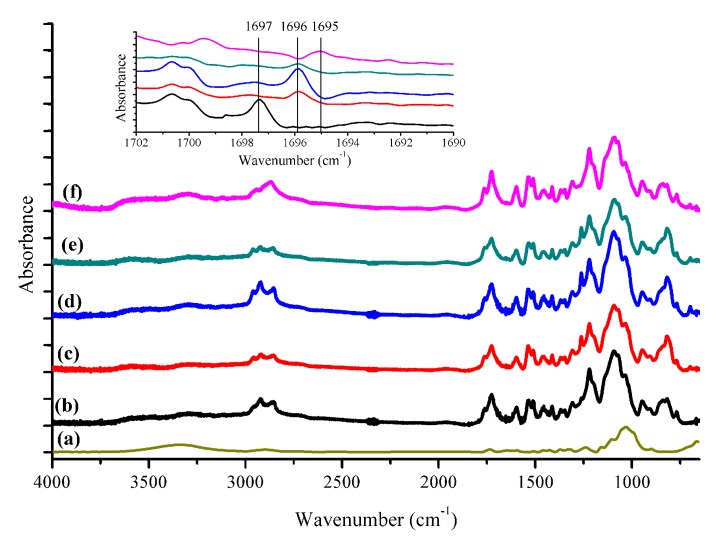
ATR-FTIR spectra of (**a**) RJF; (**b**) ASKLTPU30P; (**c**) RJF/ASKLTPU30 10/90; (**d**) PJF200/ASKLTPU30 10/90; (**e**) PJF250/ASKLTPU30 10/90; and (**f**) PJF300/ASKLTPU30 10/90 (inset graph: ATR-FTIR spectra in the range of 1702–1690 cm^−1^).

The chemical nature of ASKLTPU30P and JF-reinforced ASKLTPU was investigated using FTIR spectroscopy analysis, as shown in Figure 2. The main FTIR peaks are assigned for functional groups in Table S1 [48,56,65,66,67]. A broad peak appeared in the range of 3500–3150 cm^−1^ (N–H stretching), a peak around 1600 cm^−1^ (N–H bending), and a peak around 1550–1500 cm^−1^ (Amide II) were derived from urethane bonds. The peaks that appeared at 2950–2850 cm^−1^ were assigned to CH_2_ stretching of mainly PEG components. The peaks of carbonyl groups derived from both the acetate groups of ASKL and the urethane bonds appeared at around 1760, 1726, and 1697 cm^−1^. Due to the abundance of carbonyl groups, a relatively intensive peak appeared at 1726 cm^−1^, which as assigned to non-bonded carbonyl stretching. Interestingly, a relatively weak peak at 1697 cm^−1^ (ASKLTPU), assigned to H-bonded carbonyl stretching, slightly shifted to 1696 and 1695 cm^−1^ via the incorporation of fiber or low-temperature pyrolysis. Despite the relatively weak intensity of the peaks, the phenomenon implied the potential formation and increase of hydrogen bonding between JF and ASKLTPU via fiber incorporation and the low-temperature pyrolysis [65,68]. The increase of hydrogen bonding might have affected the interfacial bonding of JF and ASKLTPU

### 3.2. Morphological Aspects and Interfacial Bonding

**Figure 3:**
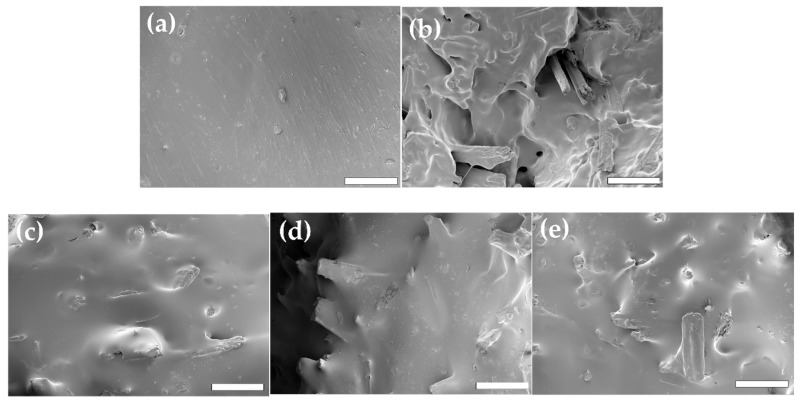
SEM images of the fracture surfaces of (**a**) ASKLTPU30; (**b**) RJF/ASKLTPU30 10/90; (**c**) PJF200/ASKLTPU30 10/90; (**d**) PJF250/ASKLTPU30 10/90; and (**e**) PJF300/ASKLTPU30 10/90 (×300, scale bar: 200 μm).

Figure 3a–e show the tensile fracture surface morphology of ASKLTPU30P and JF-reinforced ASKLTPU with a fiber content of 10 wt %. In Figure 3b, there are holes and noticeable gaps between the fibers and the polymer matrix in RJF/ASKLTPU30 10/90. Fiber pullout at fracture was also observed, which indicates easy debonding of the fiber-matrix interface during the tensile test. These mean relatively a weak bonding between RJF and ASKLTPU30 of RJF-reinforced ASKLTPU. In Figure 3c–e, however, the good wetting aspect of reinforcement by the matrix and the absence of agglomeration were observed in PJF200/ASKLTPU30 10/90, PJF250/ASKLTPU30 10/90, and PJF300/ASKLTPU30 10/90. Fiber pullout at fracture was rarely observed, which implied that the stress transfer from the fiber to the matrix took place efficiently. The morphological aspects showed that low-temperature pyrolysis of the jute fiber improved the interfacial bonding of the jute fiber and the ASKLTPU matrix. The removal of non-cellulosic compounds by the low-temperature pyrolysis was expected to increase the interfacial bonding between the JF and the ASKLTPU matrix, as shown in polypropylene (PP) composites with PJFs [30].

In order to investigate interfacial bonding between JF and ASKLTPU, the interfacial shear strength (IFSS) was obtained from a single-fiber pull-out test (Table 1). The interfacial shear strength of PJF200/ASKLTPU30, PJF250/ASKLTPU30, and PJF300/ASKLTPU30 increased by 19%, 39%, and 52%, respectively, in comparison to RJF/ASKLTPU30. Therefore, the interfacial bonding between JF and ASKLTPU increased via low-temperature pyrolysis, and it increased as the maximum pyrolysis temperature increased.

### 3.3. Mechanical Properties

**Figure 4:**
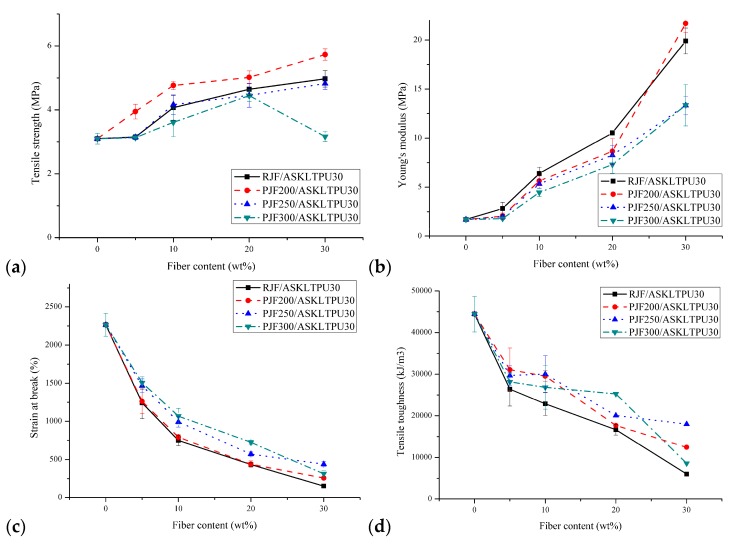

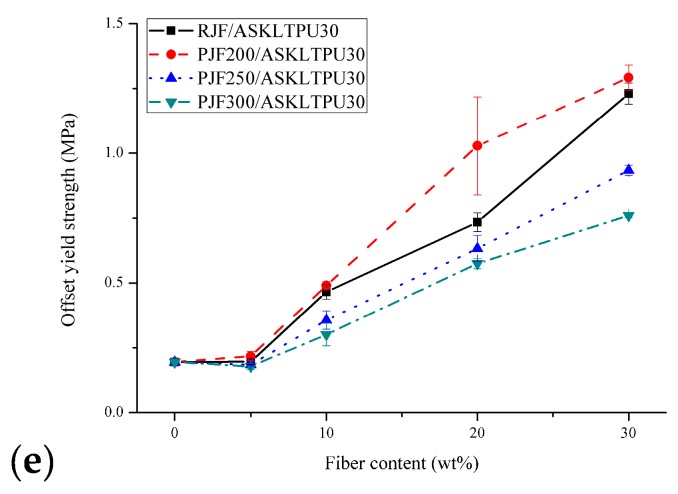
Mechanical properties of JF-reinforced ASKLTPU:(**a**) Tensile strength; (**b**) Young’s modulus; (**c**) strain at break; (**d**) tensile toughness; and (**e**) offset yield strength.

Figure 4 and Table S2 show the mechanical properties of JF reinforced ASKLTPU from tensile tests with its fiber content and maximum pyrolysis temperature of PJFs. Tensile strength, Young’s modulus, strain at break, tensile toughness, and offset yield strength were measured and are shown. 

The tensile strength ranged between 3.1 and 5.6 MPa. The tensile strength increased as the fiber content increased due to the fiber-reinforcement effect of PJFs. Exceptionally, there was a significant decrease in tensile strength on PJF300/ASKLTPU30 30/70. It might be caused by the weakness of PJF300 discussed below, which might strongly affect the tensile properties of the composite when the fiber content reached 30 wt %. At a fiber content of 5 wt %, there was no significant change in tensile strength except PJF200/ASKLTPU30 5/95 because properties of the matrix predominantly determined the properties of composites due to a relatively low reinforcement content. The low-temperature pyrolysis of jute fiber also affected the tensile strength of JF-reinforced ASKLTPU. The low-temperature pyrolysis was expected to enhance the strength of JF-reinforced ASKLTPU due to the stronger interfacial bonding shown in previous morphological aspects; however, the tensile strength of JF reinforced ASKLTPU decreased as the maximum pyrolysis temperature of PJF increased. This might be due to the decrease of tensile strength of PJFs as the maximum pyrolysis temperature increased. The tensile strength of RJF, PJF200, PJF250, and PJF300 were 0.973, 0.877, 0.461, and 0.119 N/tex, respectively, in previous work [30]. Especially, however, PJF200 reinforced ASKLTPU30 showed higher tensile strength (3.95–5.73 MPa) than RJF-reinforced ASKLTPU30 (3.15–4.97 MPa). The tensile strength of PJF200/ASKLTPU30 30/70 reached 5.73 MPa, which was an increase of 15% compared to RJF/ASKLTPU30 30/70. This might be due to the slightly weaker tensile strength of PJF200 in comparison with RJF, while those of PJF250 and PJF300 were significantly weaker than RJF. In the case of PJF200-reinforced ASKLTPU30, the enhancement effect of stronger interfacial bonding between PJF and ASKLTPU might have surpassed the negative effect from the weak properties of PJFs.

Young’s modulus ranged between 1.70 and 19.8 MPa. It increased significantly as the fiber content increased, while it decreased as the maximum pyrolysis temperature increased. Further discussions of Young’s modulus regarding the fiber aspect ratio frequency dispersion and prediction from related model will be discussed in the following section. The strain at break ranged between 170 and 2270%. It increased significantly as the fiber content decreased, and it increased as the maximum pyrolysis temperature increased. The increase of Young’s modulus and decrease of strain at break with fiber content increase was due to the reinforcement effect of stiff PJFs as the fiber component with high modulus and low elongation (jute fiber: 2.3% [30]). The gradual increase in modulus and decrease in elongation with the increase in fiber content were typical aspects of short, fiber-reinforced elastomers, as shown in many studies [42,51,69,70].

Tensile toughness of the material was measured from the area under the stress-strain curve and is typically influenced largely by ductility. The tensile toughness of JF-reinforced ASKLTPU also showed similar tendency aspects to that of strain at break, which indicated the ductility of JF-reinforced ASKLTPU.

The offset yield strength of JF-reinforced ASKLTPU was used to compare the required strength for initiation of appreciable deformation. The offset at 0.2% strain was used for measuring the offset yield strength in order to measure the strength sensitively. The offset yield strength showed a similar trend to the tensile strength or Young’s modulus because the stress at low strain was significantly affected by Young’s modulus. As the fiber content increased, the offset yield strength significantly increased, except at a low fiber content of 5 wt %. The offset yield strength of PJF200- and RJF-reinforced ASKLTPU were similar, while that of PJF250- and PJF300-reinforced ASKLTPU showed a lower yield strength.

In summary, the tensile strength, Young’s modulus, and offset yield strength of JF-reinforced ASKLTPU composites increased as the fiber content increased and the maximum pyrolysis temperature of PJF decreased with the costs of decreases in ductility and tensile toughness. Although increase in the fiber-matrix interfacial bonding was expected due to the low-temperature pyrolysis of jute fibers, the weakness of PJF itself affect negatively on strength of the JF-reinforced ASKLTPU. However, PJF200-reinforced ASKLTPU showed the highest tensile strength due to the slightly weaker strength of PJF200 and an increase in interfacial bonding. From the investigations, the mechanical properties of JF-reinforced ASKLTPU composites could be tunable for possible applications by changing the fiber content and maximum pyrolysis temperature of PJF.

### 3.4. Prediction of Elastic Modulus

**Figure 5:**
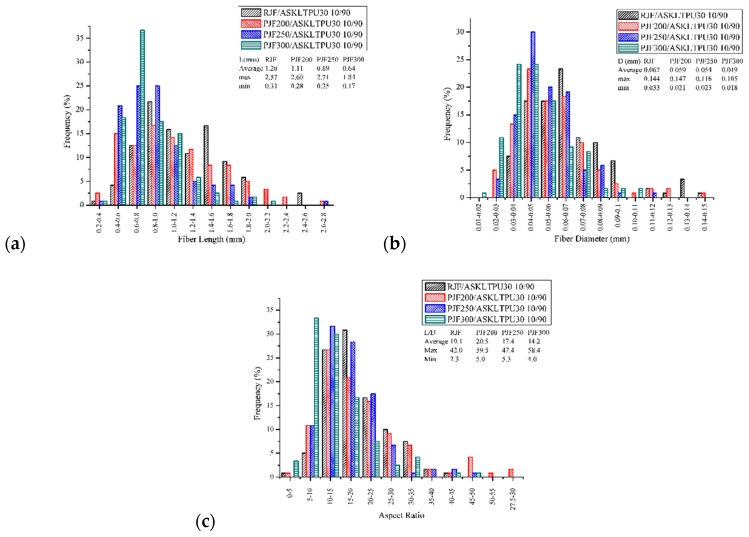
Variations of the (**a**) length; (**b**) diameter; and (**c**) aspect ratio of JFs in JF-reinforced ASKLTPUs.

The variations of length, diameter, and aspect ratio of JFs in JF-reinforced ASKLTPU with 10 wt % of fiber content was measured in order to investigate the effect of low-temperature pyrolysis of jute fibers on the JF-reinforced ASKLTPU (Figure 5). The length and diameter of jute fibers decreased using low-temperature pyrolysis, and further decreased as the maximum pyrolysis temperature increased. The fiber length decreased from its preprocessed length (10 mm in this experiment) because of the well-known devastating effects of the processing technique [40]. The higher degree of fiber length degradation of PJFs was believed to result from its inferior mechanical properties compared to raw jute fiber. In the case of fiber diameter, low-temperature pyrolysis caused the removal of non-cellulosic compounds of jute fibers [30] and probable isolation or exfoliation of individual fibers [40]. The diameter of jute fibers decreased as the maximum pyrolysis temperate increased because the degree of the removal and the probable isolation or exfoliation would increase. Therefore, the aspect ratio (length/diameter of fiber) of PJFs were comparable to RJF in spite of further decrease in fiber length. The average aspect ratio of PJF200 (20.5) was even higher than that of RJF (19.1), while those of PJF250 and PJF300 were 17.4, and 14.2, respectively.

There are mathematical models for the prediction of mechanical properties including Young’s modulus of reinforced composites to anticipate properties or applications of the composite materials. The rule of mixture (ROM) [40,71], Halpin-Tsai’s equation [40,71,72], the equation from Cox and Krenchel [41,73,74,75], and other empirical equations [76] were used for the prediction of Young’s modulus on composites.

The rule of mixture is basic and the simplest model. It is calculated based on the volume fraction of each component of the composite:

(3) $$E_{upper}=E_{f}v_{f}+E_{m}v_{m}=E_{f}v_{f}+E_{m}\left(1-v_{f}\right)$$

(4) $$E_{lower}=\;\frac{E_{f}E_{m}}{E_{f}v_{m}+E_{m}v_{f}}=\;\frac{E_{f}E_{m}}{E_{f}\left(1-v_{f}\right)+E_{m}v_{f}}$$

*E_upper_* means the upper bound of the modulus, which corresponds to longitudinal loading, while *E_lower_* means the lower bound of the modulus corresponding to transverse loading. *E* and *v* are the modulus and volume fraction of each component, respectively, while *f* and *m* are designated to the fiber and matrix component, respectively [40,71].

Halpin-Tsai’s equation is based on “self-consistent micromechanics solutions” developed by Hill [37,46] and used in order to predict the modulus for various orientations and geometries of reinforcement:

(5) $$\frac{p_{c}}{p_{m}}=\frac{1+\zeta \eta v_{r}}{1-\eta v_{r}}\;$$

(6) η=(prpm)−1(prpm)+ζ

where *p* is a composite property like modulus, *ζ* is the filler geometry reinforcement parameter, *v* is the volume fraction; *r, m*, and *c* are designated to reinforcement, matrix, and composite, respectively.

In the case of fiber, the filler geometry reinforcement parameter, *ζ*, is 2(*l*/*d*) for *E_11_* (longitudinal) and 2 for *E_22_* (transverse), while *l*, *d*, and *l/d* are designated for length, diameter, and aspect ratio of the fiber, respectively. In the case of randomly-oriented (in-plane, 2-D) discontinuous fiber composites or lamina, the predicted Young’s modulus was calculated using:

(7) $$E_{random}=\frac{3}{8}E_{11}+\frac{5}{8}E_{22}$$

The *E_random_* from the Halpin-Tsai equation could be used for a prediction study of JF-reinforced ASKLTPU with the measured aspect ratios of RJF and PJFs as the reinforcement [71,72].

The Cox-Krenchel approach is based on the equation derived from Cox’s “shear-lag” analysis regarding the effect of orientation of the fiber [73] and the orientation efficiency factor of Krenchel [41], as summarized by Folkes [74,75]. Young’s modulus of the composite is predicted as follows:

(8) $$E_{c}=\eta_{o}\eta_{L}v_{f}E_{f}+V_{m}E_{m}$$

where *η_o_* is the orientation efficiency factor and *η_L_* is the fiber length efficiency factor. The fiber length efficiency factor *η_L_* is calculated using:

(9) ηL=1−tanh(Βl2)(Βl2)

(10) B=(2πGmEfAfln(Rrf))12

where *l, A_f_, G_m_, R,* and *r_f_* are the length of the fiber, cross-sectional area of fiber, shear modulus of the matrix, the mean separation of the fibers normal to their length, and radius of fiber, respectively. *R* is often calculated from equations using the assumption of hexagonally packed fibers or square-packed fibers [41,73,74,75].

(11) $$v_{f}=\frac{2\pi r^{2}}{R^{2}\sqrt{3}}\;(\mathrm{hexagonally}\;\mathrm{packed}),\;v_{f}=\frac{\pi r^{2}}{R^{2}}\;(\mathrm{square}\;\mathrm{packed}).$$

The orientation efficiency factor *η_o_* is given using:

(12) $$\eta_{0}=\underset{n}{\sum }a_{n}\cos^{4}\phi_{n},\;\mathrm{where}\;\underset{n}{\sum }a_{n}=1$$

where *a_n_* is the fraction of fibers with orientation angle *Φ_n_* with respect to the reference axis [41,74].

In the case of JF-reinforced ASKLTPU, *η_o_* was set to 0.375 for random in-plane orientation (2-D random) [40,41] and the fiber components were assumed to be square-packed. The measured length, diameter, and aspect ratio of PJFs were used for the calculation.

**Figure 6:**
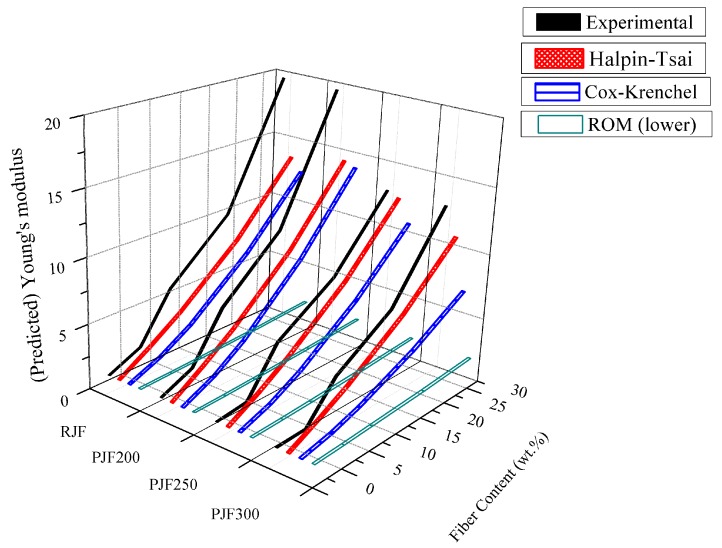
Experimental and predicted Young’s modulus of JF-reinforced ASKLTPU modelled using Halpin-Tsai, Cox-Krenchel, and the rule of mixture (lower).

Young’s modulus of JF-reinforced ASKLTPU modelled using Halpin-Tsai, Cox-Krenchel, and ROM was calculated in order to predict Young’s modulus and compare experimental results (Figure 6). ROM for the upper limit was excluded due to an extraordinarily high predicted value (2–15 GPa) calculated from the large difference of moduli of the fiber and elastomeric matrix. ROM for the lower limit was used to find the lower bound of the predicted Young’s modulus. It ranged from 1.8 MPa to 2.4 MPa. The predicted Young’s modulus of JF-reinforced ASKLTPU from both Halpin-Tsai and Cox-Krenchel showed similar trends in the range of calculation: fiber contents from 5 wt % to 30 wt %. Overall, JF-reinforced ASKLTPU with a fiber content of 10 wt % showed a similar or slightly higher experimental modulus in comparison to the predicted value. Meanwhile, JF-reinforced ASKLTPU with fiber contents of 5 wt % showed a lower experimental value and JF-reinforced ASKLTPU with fiber contents of 20 and 30 wt % showed a higher experimental Young’s modulus in comparison to predicted values. The trend in difference of experimental and predicted Young’s modulus is believed to result from multiple reasons. JF-reinforced ASKLTPU as a short-fiber-reinforced elastomer might have different features from other biocomposites due to a TPU matrix with elastomeric mechanical properties and characteristics of microphase separation. The modifying factors might be needed to predict the modulus precisely as shown in another study of reinforced rubber [77]. Moreover, the length, diameter, and aspect ratio of JF used for the prediction were measured from JF-reinforced ASKLTPU with a fiber content of 10 wt %. For JF-reinforced ASKLTPU with other fiber contents, the actual value of the aspect ratio after the processing could differ from the value used for prediction [74]. In addition, the increased interfacial bonding might affect the experimental Young’s modulus in the case of PJF-reinforced TPU [78]. Thus, Young’s modulus of JF-reinforced ASKLTPU might be predicted from the Halpin-Tsai equation and Cox-Krenchel equation; however, the difference from the experimental value should be considered.

### 3.5. Dynamic Mechanical Thermal Analysis (DMA)

The dynamic mechanical thermal analysis of JF-reinforced ASKLTPU was performed to investigate the stiffness and the damping factor of the material under dynamic conditions as a function of temperature. PJF250-reinforced ASKLTPU with 10, 20, and 30 wt % of fiber loading was selected to measure and compare with ASKLTPU30P and RJF-reinforced ASKLTPU. The storage modulus (*M*′), loss modulus (*M*″), loss tangent (tanδ), and specific values including the glass transition temperature (*T*_g_) from the compression mode of DMA are shown in Figure S2 and Table 2.

As shown in Figure S2a, the storage modulus of JF-reinforced ASKLTPU was higher than ASKLTPU30P and increased as the fiber content increased because of the reinforcement effect of short fibers. The trend of variation of storage modulus and loss modulus at 25 °C shown in Table was almost in good agreement with that of results from the tensile test in spite of the different mode of experimental conditions. The modulus of JF-reinforced ASKLTPU at 25 °C was higher than that of ASKLTPU30P, and an increase of fiber loading up to 30 wt % affected the increase of storage modulus of JF-reinforced ASKLTPU. RJF-reinforced ASKLTPU showed a higher modulus than PJF250-reinforced ASKLTPU, which was similar to the trend of Young’s modulus from tensile tests, with the exception of PJF250/ASKLTPU30 30/70.

The onset of the decrease of storage modulus or the peak of loss modulus and loss tangent around 0 °C indicated the glass transition of the compatible soft and hard segment mixtures of the ASKLTPU matrix [56]. In Table 2, the glass transition temperature (*T*_g_) from the peak of loss tangent and from the peak of loss modulus are shown. Although the higher *T*_g_ from the loss tangent is also valid and widely used for determining *T*_g_, the *T*_g_ from the loss modulus was more appropriate for determining *T*_g_ in order to denote the initial drop of storage modulus, especially on JF-reinforced ASKLTPU with a broad range of thermal transition region [79]. The *T*_g_ shifted toward a higher temperature via the incorporation of fiber.

In the case of the loss tangent of ASKLTPU and JF-reinforced ASKLTPU as an index of energy dissipation, the maximum loss tangent value decreased and the width of loss tangent peak broadened as the fiber content increased (Figure S2c and Table 2). The phenomenon was attributed to the reduced mobility of the matrix via the reinforcement effect of fibers [42,43]. The asymmetric broader range of thermal transition also occurred from various molecular relaxation behaviors with additional energy dissipation derived from various fiber-matrix interfaces [38,52,72]. In the case of PJF250-reinforced ASKLTPU, the maximum loss tangent was lower, and the loss tangent peak was broader than RJ- reinforced ASKLTPU with the same fiber content. It is known that the damping at the interfaces was lower when the interface adhesion was higher, which might be due to a higher reinforcement effect or better relaxation occurring at a higher volume of stronger interfaces [52,72,80,81]. The loss tangent peak also indicated that PJF250 had a stronger interface adhesion with ASKLTPU in comparison to RJF. The higher storage modulus and lower loss tangent of JF-reinforced ASKLTPU with different fiber loadings showed a similar trend to other studies of short-fiber-reinforced elastomers [41,42,43,52,80].

In addition, the onset of the decrease or the peak of the storage modulus and loss tangent appearing over the temperature of 150 °C indicated the transition of a microphase-separated hard domain of thermoplastic polyurethane matrix [56]. Due to thermoplastic behaviors over the transition and low storage modulus, ASKLTPU and JF-reinforced ASKLTPU were able to reprocess over the temperature of 150 °C like thermoplastics. 

### 3.6. Thermal Transition Behavior

**Figure 7:**
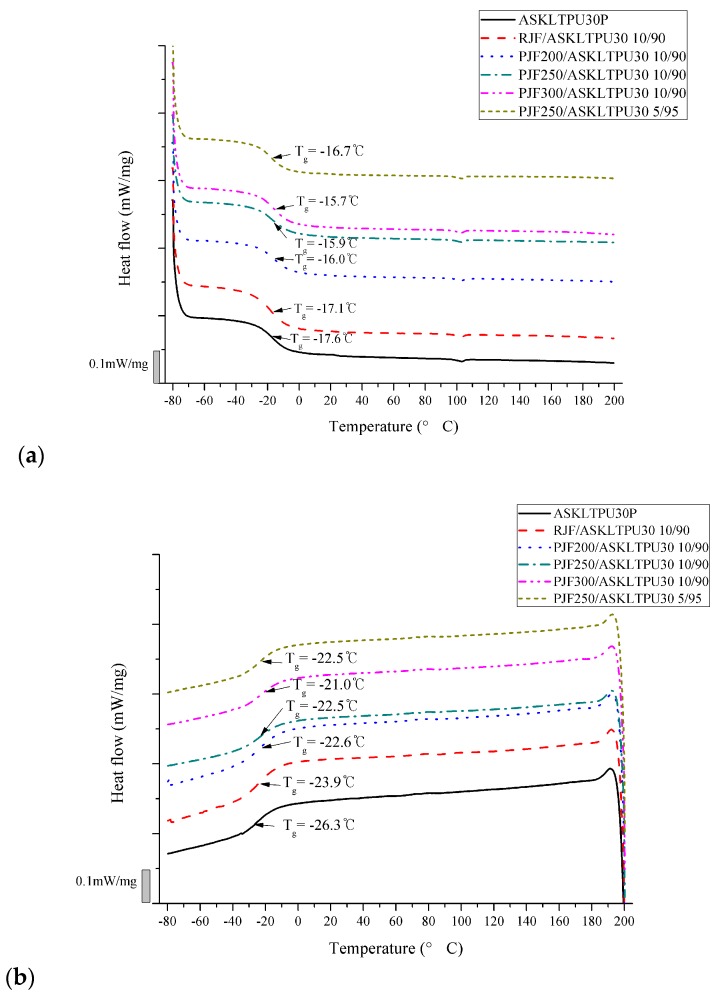
DSC thermograms of ASKLTPU30P and JF-reinforced ASKLTPU30: (**a**) the second heating curve; and (**b**) the first cooling curve.

The thermal transition behavior of JF-reinforced ASKLTPU was investigated using differential scanning calorimetry (DSC) and dynamic mechanical thermal analysis (DMA) above. Figure 7 shows the second heating curve and the first cooling curve of the DSC thermograms of ASKLTPU30P and JF-reinforced ASKLTPU30. As mentioned above in discussions of DMA, the glass transition of the compatible soft and hard segment mixtures of the ASKLTPU matrix occurred at the temperature in the range of −17.6 and −15.7 °C (second heating curve) or in the range of −26.3 and −21.0 °C (first cooling curve). There was a difference in *T*_g_ from DSC and *T*_g_ from DMA (from −11.7 to −2.3 °C) because the glass transition is a kind of kinetic transition and could differ with the measuring conditions and techniques; *T*_g_ of DSC was measured from heat capacity while that of DMA was measured from relaxation [79]. Nevertheless, *T*_g_ from DSC and *T*_g_ from DMA had a similar trend of change due to fiber loading and low-temperature pyrolysis. Both *T*_g_s became higher via the incorporation of fiber, and the tendency of increasing *T*_g_ was observed as the fiber content of JF-reinforced ASKLTPU increased. Low-temperature pyrolysis of jute fibers also slightly increased the *T*_g_ of JF-reinforced ASKLTPU. The increase of *T*_g_ might have occurred from the reduced mobility of the matrix via the reinforcement effect of fibers. A similar trend has been shown in other studies about elastomers reinforced by short-fiber- or nano-reinforcement [43,52,69,76,82], although other studies showed consistency [41,80] or a decrease [37,42,46] of *T*_g_ from an increase of reinforcement loading or modification of reinforcement.

There was no significant thermal transitions except the glass transition in DSC curves. The transition of the microphase-separated hard domain of thermoplastic polyurethane matrix, which is shown in DMA results, was too weak to analyze like the previous work [56]. No significant crystallization behavior occurred during the cooling and heating stages. In Figure S3, X-ray diffraction (XRD) analysis also supported the amorphous nature of the ASKLTPU and JF-reinforced ASKLTPU. A broad peak (halo) near the degree of 2θ = 21.5° might be assigned to the partially ordered structure of ASKL-MDI hard segment domain [68], which corresponds to a relatively weak intensity of ASKLTPU10 with a low content of the hard domain. The short RJF and PJF did not induce crystallization behavior of the ASKLTPU matrix, so the thermal transition related to crystallization behavior did not occur in the DSC curve. The similar results were also obtained at many kinds of polyurethane with an inherent amorphous nature including castor oil-based PU [83] and polyester-type TPU [84], although induced hard phase crystallization of TPU via cellulose nanocrystals also occurred in other study [45]. 

### 3.7. Thermal Stability

**Figure 8:**
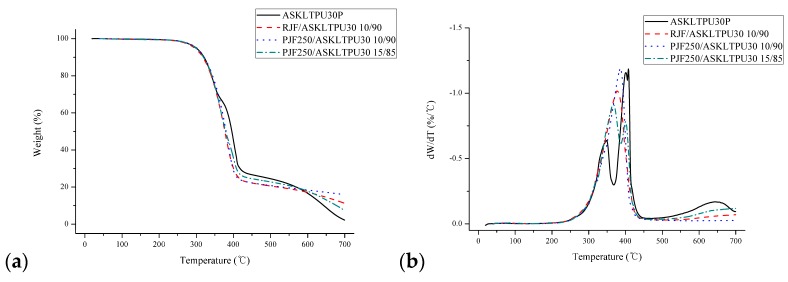
(**a**) TGA and (**b**) derivative of the TGA (DTG) results of ASKLTPU30P.

Thermogravimetric analysis (TGA) is often used in order to rank the relative thermal stability of a group of samples and to study degradation behavior of the samples [79]. Figure 8 and Table 3 shows the results of TGA and the derivative of the TGA (DTG) of ASKLTPU30P and JF-reinforced ASKLTPU. *T*_−1%_, *T*_−5%_, and *T*_−10%_ indicated the temperatures at which 1, 5, and 10% of weight decreased. *T*_d_ indicated the maximum decomposition temperature while *T*_peak_ indicated the temperature at which the peak in DTG curves occurred, and the symbol ‘~’ identifies the shoulder peaks.

The DTG curve of ASKLTPU30P showed two thermal decomposition steps. The first step around 348 °C indicated the degradation of hard segment based on ASKL and decomposition of MDI with the breaking urethane bonds, while the second consecutive steps around 400 °C indicated the chain-scission of the soft segment based on PEG1000 [39,42,56,83]. In the previous work, three DTG peaks of raw jute fiber appeared at approximately 261 °C (shoulder), 354 °C, and 391 °C (shoulder), which correlated with the hemicellulose, cellulose, and lignin components of jute fibers, respectively [30,31].

The DTG curves of RJF/TPU30 10/90 and PJF250/TPU30 10/90 showed one wide peak. It was estimated that the main thermal decomposition of jute fibers, the cellulose component at around 354 °C, overlapped with that of ASKLTPU30P. Therefore, it was shown as one wide thermal decomposition; however, in reality, there were different thermal decompositions in the peak. In the case of PJF250/TPU30 15/85, the larger portion of the PJF200 component contributed more to the thermal profile of the JF-reinforced ASKLTPU, so its DTG peak occurred at 366.9 °C and 400.5 °C.

As shown in Table 3, the thermal stability of ASKLTPU30P was better than that of RJF/ASKLTPU 10/90 due to relatively low thermal stability of jute fibers. It has often been observed that the incorporation of natural fibers has no positive or negative effect on thermal stability of natural fiber reinforced composites and elastomers due to the relatively low thermal stability of natural fibers to the matrix [70,71,85]. However, PJF250-reinforced ASKLTPU showed better thermal stability than RJF-reinforced ASKLTPU as a result of low-temperature pyrolysis, which removed non-cellulosic compounds with a low decomposition temperature [30]. PJF250/ASKLTPU30 10/90 even showed better thermal stability than ASKLTPU30P. In addition, up to the temperature of 350 °C, *T*_−1%_, *T*_−5%_, *T*_−10%_, and residual weight indicated the JF-reinforced ASKLTPU had better thermal stability considering the sum of the components’ weight fraction solely. This might have resulted from the modified fiber-matrix interface or structural stability. Overall, the low-temperature pyrolysis of the jute fiber enhanced the thermal stability of the JF-reinforced ASKLTPU.

### 3.8. Water Absorption

**Figure 9:**
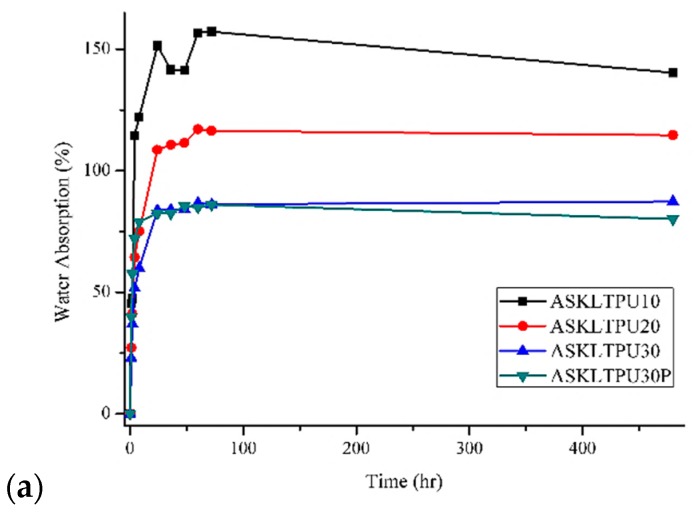

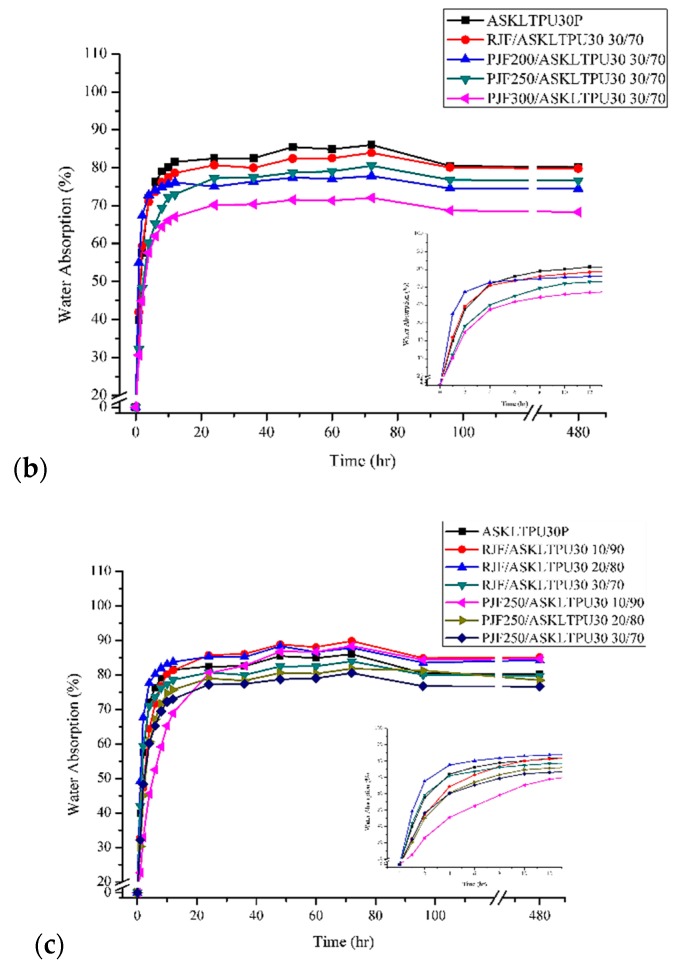
Water adsorption of (**a**) ASKLTPUs; (**b**) and (**c**) JF-reinforced ASKLTPU with different maximum pyrolysis temperature or different fiber content. (Inset graph: 0–12 h).

The water absorption behavior of ASKLTPU and JF-reinforced ASKLTPU was tested and evaluated for potential applications (Figure 9). As shown Figure 9a, the water absorption behavior of ASKLTPU increased as the ASKL content and hard segment component of ASKLTPU decreased. ASKL content. In the case of ASKLTPU10 and ASKLTPU20, the increased fraction of the PEG component as the soft segment of ASKLTPU increased the water absorption of ASKLTPU. In addition, the processing procedure on ASKLTPU30 had little effect on the water absorption properties in comparison with ASKLTPU30 and ASKLTPU30P. Figure 9b shows the water absorption behavior of ASKLTPU30P and JF-reinforced ASKLTPU with 30 wt % of fiber content. All JF-reinforced ASKLTPU with a fiber content of 30 wt % had lower water absorption at equilibrium than ASKLTPU30P. Low-temperature pyrolysis on jute fibers lowered the water absorption of JF-reinforced ASKLTPU, and the degree of decrease in water absorption increased as a maximum pyrolysis temperature of low-temperature pyrolysis increased. The increased compatibility and interfacial bonding of PJF and ASKLTPU30 lowered the water absorption of JF-reinforced ASKLTPU [54,68]. The decrease of hydrophilicity via the removal of non-cellulosic materials of jute fibers after low-temperature pyrolysis also affected the decrease of water absorption of JF-reinforced ASKLTPU [30]. Figure 9c shows the water absorption behavior of ASKLTPU30P, RJF-reinforced ASKLTPU, and PJF250-reinforced ASKLTPU with different fiber contents. In general, the incorporation of lignocellulosic natural fibers as the reinforcement of elastomers or composites increased the water absorption because of hydrophilic nature of lignocellulosic natural fibers [52,54]. In the case of JF-reinforced ASKLTPU, RJF/ASKLTPU30 10/90, RJF/ASKLTPU30 20/80, and PJF250/ASKLTPU30 10/90 showed slightly higher water absorption at equilibrium due to the hydrophilic aspect of lignocellulosic natural fibers. At higher fiber contents, however, RJF/ASKLTPU30 30/70 showed similar water absorption to ASKLTPU while PJF250/ASKLTPU30 20/80 and PJF250/ASKLTPU30 30/70 showed lower water absorption at equilibrium than ASKLTPU. The decrease of water absorption in equilibrium was estimated to result from the physically dense structure derived from fiber loading and increase in hydrophobicity of fibers in the case of PJF250. In addition, before 12 h of soaking, water adsorption increased as the fiber loading increased in the case of JF-reinforced ASKLTPU. It is supposed that more fiber exposed to the surface of the JF-reinforced ASKLTPU of the higher fiber content increased the water absorption of the early stage; meanwhile, the higher fiber content decreased the water absorption in equilibrium via a physically denser structure. In summary, the water absorption of JF-reinforced ASKLTPU decreased by the low-temperature pyrolysis and changed by combination of inherent hydrophilic nature, reduced the hydrophilicity via low-temperature pyrolysis, and physically dense structure from fiber loading.

### 3.9. Degradation Test

The degradation test of ASKLTPU and JF-reinforced ASKLTPU was performed with an acceleration of *Aspergillus* fungi, whose *spp.* produces esterase or lignin peroxide with the potential of lignin degrading ability [86,87,88]. The weight loss and change in mechanical properties after the fungal degradation test were measured to investigate deterioration via the degradation and biodegradability of samples.

**Figure 10:**
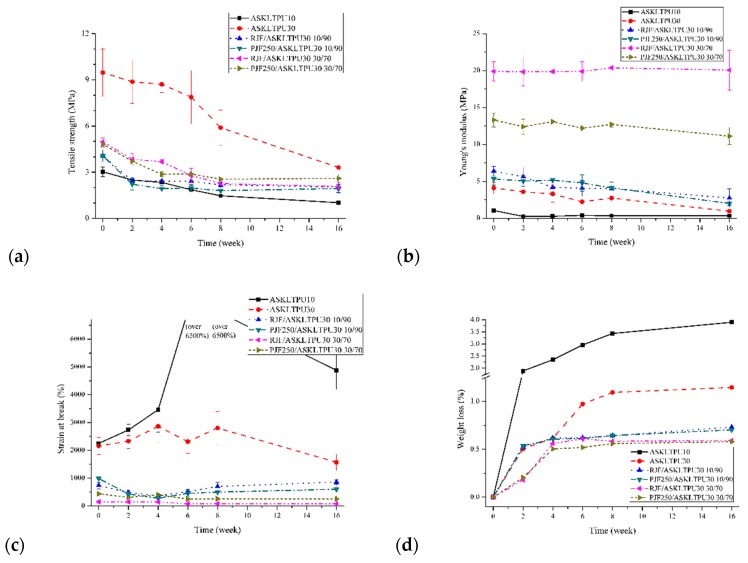
The weight loss and change in mechanical properties of ASKLTPU and JF-reinforced ASKLTPU during the fungal degradation test: (**a**) weight loss; (**b**) tensile strength; (**c**) Young’s modulus; and (**d**) strain at break.

Figure 10a shows the weight loss of ASKLTPU and JF-reinforced ASKLTPU. After 16 weeks of biodegradation, the weight loss of ASKLTPU10 and ASKLTPU30 were 3.90% and 1.15%, respectively. The higher weight loss of ASKLTPU10 than ASKLTPU30 implied the faster degradation of the PEG component of ASKLTPU. The hard domain based on the ASKL component of ASKLTPU might show slower degradation due to the physical crosslinking effects of the hard domain of thermoplastic of polyurethane. The similar trend of increase in the biodegradability by increasing the soft segment content was also observed in another study [89]. Moreover, the weight loss of the JF-reinforced ASKLTPU ranged from 0.58% to 0.73%, which was lower than the ASKLTPU30 matrix. Although the incorporation of the natural fiber increased the biodegradability in other studies of biocomposites [90,91], the biodegradability of JF-reinforced ASKLTPU decreased as fiber content increased. This might have been due to the faster degradation rate of ASKLTPU based on ASKL and PEG than that of jute fiber. The effect of fungal degradation, low crystallinity of ASKLTPU, and a relatively susceptible soft segment of ASKLTPU would affect the faster degradation rate [90]. Thus, the incorporation of fiber retarded the biodegradation rate and lowered the weight loss at 16 weeks of biodegradation, similar to the results from other polyurethane-based biocomposites [68]. The low-temperature pyrolysis of jute fiber, however, had little effect on the degradation of JF-reinforced TPU, though the weight loss of PJF-reinforced ASKLTPU was slightly less than RJF-reinforced ASKLTPU. Because of the increased interfacial bonding and removal of relatively liable non-cellulosic compounds, PJF-reinforced ASKLTPU showed less weight loss. However, as degradation of the ASKLTPU matrix dominated the degradation of JF-reinforced ASKLTPU, the degree of difference of degradation might be limited.

Figure 10b–d shows the change in the mechanical properties of ASKLTPU and JF-reinforced ASKLTPU. In Figure 10b, the tensile strength of ASKLTPU and JF-reinforced ASKLTPU gradually decreased as the degradation test was performed. After 16 weeks of the degradation test, the tensile strength of ASKLTPU10 and ASKLTPU30 decreased to 34% and 35%, respectively, of the original ones. In the case of JF-reinforced ASKLTPU, the tensile strength of RJF/ASKLTPU30 10/90, PJF250/ASKLTPU30 10/90, RJF/ASKLTPU30 30/70, and PJF250/ASKLTPU30 30/70 decreased to 50%, 47%, 42%, and 54%, respectively, after 16 weeks of the degradation test. It was noticed that the tensile strength of unreinforced ASKLTPU decreased more during the degradation test in comparison to JF-reinforced ASKLTPU. In Figure 10c, Young’s modulus also gradually decreased in the case of ASKLTPU and JF-reinforced ASKLTPU with a fiber content of 10 wt %. The JF-reinforced ASKLTPU with fiber content of 30 wt %; however, Young’s modulus remained almost constant during the 16 weeks of the degradation test. Considering the decreasing trend of tensile strength and Young’s modulus and weight loss discussed above, it is believed that the ASKLTPU matrix synthesized from ASKL, PEG, and MDI degraded and deteriorated first. Then, the jute fibers and fiber-matrix interface degraded and deteriorated at a relatively slow speed under the settings and conditions of the performed degradation test. In the case of the JF-reinforced ASKLTPU with a fiber content of 30 wt %, fibers with a relatively high content maintained their Young’s modulus in spite of matrix deterioration because the modulus of the composite was highly affected by the modulus of the fiber. In Figure 10d, the strain at break, indicative of elongation, and ductility of materials are shown and the characteristic features are to be discussed. The strain at break of ASKLTPU or JF-reinforced ASKLTPU with a fiber content of 10 wt % increased in the specific period before the final measurement at 16 weeks of degradation. The strain at break of ASKLTPU10 gradually increased before the final measurement, and even reached >6500% strain, the limit of the instruments, without breaking at 6 and 8 weeks of degradation. The strain at break of RJF/ASKLTPU30 10/90 and PJF250/ASKLTPU30 10/90 decreased first but increased after 6 weeks of degradation. This phenomenon could be explained by the plasticizing effect of degradation product related to the soft segment of ASKLTPU. The soft segment based on PEG1000 was considered to be degraded relatively fast, as discussed above. The degradation products related to PEG1000, such as oligomeric polyethylene glycol and ethylene glycol, would be produced during degradation tests and act as a plasticizer in the ASKLTPU matrix. Therefore, the strain at break of the material increased in spite of deterioration during the specific period of degradation. In the case of ASKLTPU10, the higher soft segments based on PEG resulted in a drastic increase of the strain at break. In the case of the JF-reinforced ASKLTPU with a fiber content of 30 wt %, however, the strain at break was almost retained after 6 weeks of degradation. The higher fiber content might have retarded the rate of degradation and deterioration. It also matched the results of maintained Young’s modulus discussed above. Overall, the low-temperature pyrolysis of jute fibers also showed slightly higher resistance to deterioration during biodegradation tests, and the reasons of little difference might be the same as discussed regarding the weight loss above.

## 4. Conclusions

Pyrolyzed jute fiber-reinforced acetylated lignin-based thermoplastic polyurethane with a fiber content from 5 to 30 wt % was prepared using a melt-mixing method followed by hot-press molding and characterized as short-fiber-reinforced elastomers with carbon-neutrality and biodegradability. The increase of interfacial bonding between PJF and ASKLTPU was observed using scanning electron microscopy and a single-fiber pull-out test. PJFs showed good wetting aspects to ASKLTPU and higher IFSS between ASKLTPU than that of RJF. In the case of tensile tests, tunable mechanical properties of JF-reinforced ASKLTPU were obtained. The strength and modulus of JF-reinforced ASKLTPU composites increased as the fiber content increased and the maximum pyrolysis temperature of PJF-decreased with a decrease in ductility and tensile toughness. The weakness of PJF itself negatively affected the strength of the JF-reinforced ASKLTPU in spite of the increase in the fiber-matrix interfacial bonding via low-temperature pyrolysis of jute fibers. Young’s modulus predicted using Halpin-Tsai model and Cox-Krenchel models were comparable to experimental results; however, the difference between the prediction and experimental results should be considered. Among the thermal transition temperature of JF-reinforced ASKLTPU, the glass transition temperature of the soft segment of the ASKLTPU matrix increased via fiber incorporation and low-temperature pyrolysis. The thermal stability of JF-reinforced ASKLTPU increased via low-temperature pyrolysis. The water adsorption behavior and degradation characteristics of JF-reinforced ASKLTPU was also investigated via a water adsorption test and degradation test using *Aspergillus* fungi. The characteristic degradation and deterioration behavior was shown because the PEG component as a soft segment of ASKLPTU matrix degraded and deteriorated first during the degradation test.

In conclusion, the low-temperature pyrolysis of jute fibers affected the properties of JF-reinforced ASKLTPU via increasing interfacial bonding and reducing inherent hydrophilicity of natural fibers. The properties of PJF-reinforced ASKLTPU indicated potential utilization as a bio-based short-fiber-reinforced elastomers.

## Figures and Tables

**Table 1:** Interfacial shear strength (IFSS) between JF and ASKLTPU30 measured from a single-fiber pull-out test.

Fiber/Matrix	Interfacial Shear Strength (IFSS) (MPa)
RJF/ASKLTPU30	2.32 ± 0.04
PJF200/ASKLTPU30	2.75 ± 0.02
PJF250/ASKLTPU30	3.23 ± 0.04
PJF300/ASKLTPU30	3.53 ± 0.02

**Table 2:** Specific results of dynamic mechanical analysis of JF reinforced ASKLTPU.

Samples	at 25 °C		at tanδ Peak		at *M*’’ Peak
	*M*’ (MPa)	*M*’’ (MPa)	Max. tanδ	*T*_g_ (°C) from tanδ	*T*_g_ (°C) from *M*’’
TPU30P	19.3	8.9	0.70	3.1	−11.7
RJF/ASKLTPU30 10/90	43.6	22.1	0.62	8.35	−8.5
RJF/ASKLTPU30 20/80	56.3	25.1	0.52	7.3	−7
RJF/ASKLTPU30 30/70	69.3	32.8	0.48	11.2	−7.9
PJF250/ASKLTPU30 10/90	27.7	13.5	0.51	9.4	−6
PJF250/ASKLTPU30 20/80	47.2	18.9	0.46	8.5	−2.3
PJF250/ASKLTPU30 30/70	101.8	40.4	0.44	8.15	−4.8

**Table 3:** TGA and derivative of the TGA (DTG) results of JF-reinforced ASKLTPU.

Sample	*T*_−1%_ (°C)	*T*_−5%_ (°C)	*T*_−10%_ (°C)	*T*_d_ (°C)	*T*_peak_ (°C)
TPU30P	242.0	299.7	321.1	408.2	348.4, 400.9, 408.2
Jute Fiber [30]	31.3	72.3	263.6	353.9	~260, 353.9, ~391
RJF/ASKLTPU30 10/90	235.8	295.3	318.1	377.2	377.2
PJF250/ASKLTPU30 10/90	245.6	301.2	321.7	386.5	386.5
PJF250/ASKLTPU30 15/85	242.1	298.5	319.7	366.9	~320.9, 366.9, 400.5

## Supplementary Materials

The following are available online at http://www.mdpi.com/2073-4360/10/12/1338/s1, Figure S1. (a) The scheme of single-fiber pull-out test; (b) An example of a load-extension curve from a single-fiber pull-out test (PJF250/ASKLTPU30, L = 0.8 mm), Figure S2. Dynamic mechanical thermal analysis of JF-reinforced ASKLTPU: (a) storage modulus; (b) loss modulus; and (c) loss tangent (tanδ), Figure S3. X-ray diffraction spectra of (a) ASKLTPU10; (b) ASKLTPU30; (c) RJF/ASKLTPU30 10/90; and (d) PJF250/ASKLTPU30 10/90, Table S1. Main FTIR peak assignments for JF-reinforced ASKLTPU samples, Table S2. Mechanical properties of JF-reinforced ASKLTPU samples.
