# Supplementary material for: Effect of Low-Temperature Pyrolysis on the Properties of Jute Fiber-Reinforced Acetylated Softwood Kraft Lignin-Based Thermoplastic Polyurethane
Source: Polymers (Basel). 2018 Dec 3;10(12):1338. doi: 10.3390/polym10121338 (PMC6401921; doi:10.3390/polym10121338)
Supplement: Supplementary file 1 [file polymers-10-01338-s001.pdf]

Supplementary Materials: Effect of Low-Temperature Pyrolysis on the Properties of Jute Fiber-Reinforced Acetylated Softwood Kraft Lignin-Based Thermoplastic Polyurethane

Hyun-gyoo Roh, Sunghoon Kim, Jungmin Lee and Jongshin Park

Table S1. Main FTIR peak assignments for JF-reinforced ASKLTPU samples.

Wavenumber (cm^{-1}) [41,48,57–59]	Functional Group	ASKLTPU 30P	RJF/ASKLTPU30 10/90	PJF200/ASKLTPU30 10/90	PJF250 ASKLTPU30 10/90	PJF300/ASKLTPU30 10/90
3500-3150	N–H stretching	3301	3299	3287	3291	3297
2950-2850	CH_2 stretching (PEG component)	2921, 2854	2919,2853	2920,2854	2921,2859	2922,2869
1760, 1740	C=O stretching of acetate groups	1762	1762	1762	1762	1765
1740	Non-bonded C=O stretching	1726	1726	1726	1726	1726
1700	H-bonded C=O stretching	1697	1696	1696	1696	1695
1600	N–H bending	1597	1598	1598	1598	1598
1550-1500	(C=O)NH (Amide II)	1534, 1512	1537,1512	1539, 1512	1533, 1511	1532, 1511

Table S2. Mechanical properties of JF-reinforced ASKLTPU samples.

Fiber Content (wt %)	Mechanical Properties	RJF/ASKLTPU30	PJF200/ASKLTPU30	PJF250/ASKLTPU30	PJF300/ASKLTPU30
0	Tensile strength (MPa)	3.10 ± 0.17			
	Young's modulus (MPa)	1.70 ± 0.12			
	Strain at break (%)	2265 ± 149			
	Tensile toughness (kJ/m³)	44426 ± 4275			
	Stress at offset yield (MPa)	0.19 ± 0.01			
5	Tensile strength (MPa)	3.15 ± 0.06	3.95 ± 0.23	3.14 ± 0.07	3.13 ± 0.06
	Young's modulus (MPa)	2.83 ± 0.63	2.05 ± 0.32	2.00 ± 0.24	1.79 ± 0.11
	Strain at break (%)	1240 ± 206	1261 ± 158	1467 ± 91	1507 ± 79
	Tensile toughness (kJ/m³)	26400 ± 4054	31070 ± 5262	29676 ± 2304	28153 ± 1959
	Stress at offset yield (MPa)	0.20 ± 0.01	0.22 ± 0.02	0.18 ± 0.00	0.18 ± 0.01
10	Tensile strength (MPa)	4.07 ± 0.39	4.76 ± 0.13	4.16 ± 0.3	3.61 ± 0.45
	Young's modulus (MPa)	6.4 ± 0.64	5.65 ± 0.5	5.34 ± 0.42	4.45 ± 0.41
	Strain at break (%)	749 ± 67	791 ± 40	991 ± 68	1068 ± 103
	Tensile toughness (kJ/m³)	22870 ± 2803	29513 ± 1340	30006 ± 4396	26847 ± 5261
	Stress at offset yield (MPa)	0.47 ± 0.03	0.49 ± 0.01	0.36 ± 0.04	0.3 ± 0.04
20	Tensile strength (MPa)	4.64 ± 0.18	5.02 ± 0.2	4.45 ± 0.37	4.45 ± 0.18
	Young's modulus (MPa)	10.54 ± 0.15	8.65 ± 1.31	8.26 ± 0.95	7.30 ± 0.89
	Strain at break (%)	433 ± 21	437 ± 44	571 ± 26	724 ± 27
	Tensile toughness (kJ/m³)	16674 ± 1293	17680 ± 1423	20058 ± 1358	25257 ± 1486
	Stress at offset yield (MPa)	0.73 ± 0.04	1.03 ± 0.19	0.63 ± 0.04	0.57 ± 0.02
30	Tensile strength (MPa)	4.97 ± 0.26	5.73 ± 0.18	4.82 ± 0.18	3.16 ± 0.16
	Young's modulus (MPa)	19.9 ± 1.32	21.69 ± 0.9	13.32 ± 0.95	13.35 ± 2.11
	Strain at break (%)	151 ± 9	257 ± 21	439 ± 34	313 ± 46
	Tensile toughness (kJ/m³)	6010 ± 483	12443 ± 848	18007 ± 1511	8583 ± 1451
	Stress at offset yield (MPa)	1.23 ± 0.04	1.29 ± 0.05	0.93 ± 0.02	0.76 ± 0.01

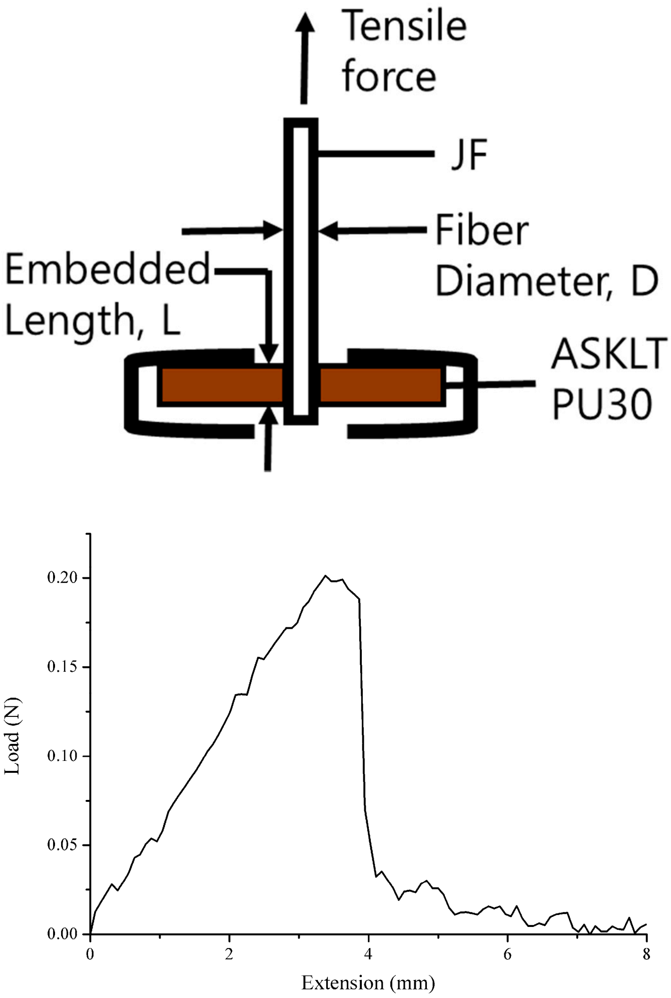

(a) (b)

Figure S1. (**a**) The scheme of single-fiber pull-out test; (**b**) An example of a load-extension curve from a single-fiber pull-out test (PJF250/ASKLTPU30, L = 0.8mm).

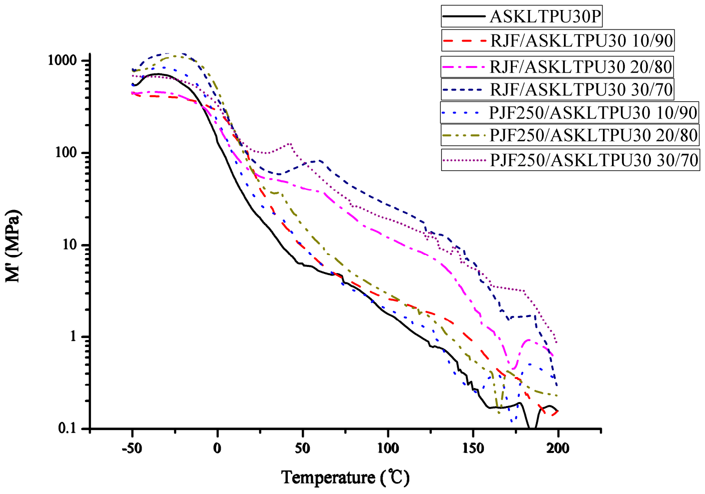

(**a**)

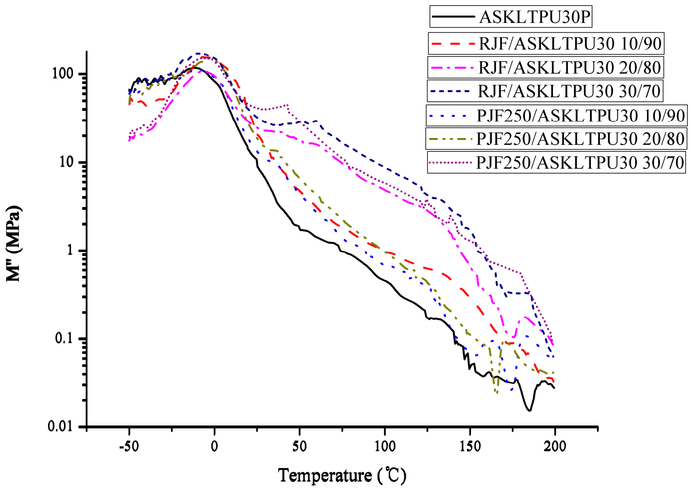

(**b**)

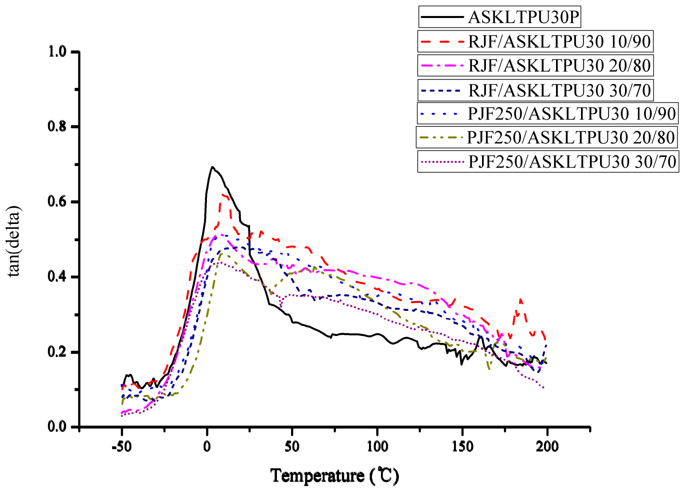

(**c**)

Figure S2. Dynamic mechanical thermal analysis of JF-reinforced ASKLTPU: (**a**) storage modulus;, (**b**) loss modulus; and (**c**) loss tangent (tanδ).

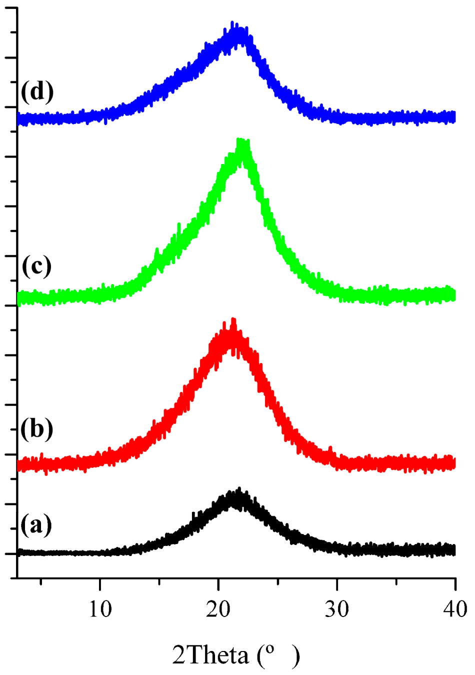

Figure S3. X-ray diffraction spectra of (**a**) ASKLTPU10; (**b**) ASKLTPU30; (**c**) RJF/ASKLTPU30 10/90, and (**d**) PJF250/ASKLTPU30 10/90.
